# Acute Mountain Sickness Following Incremental Trekking to High Altitude: Correlation With Plasma Vascular Endothelial Growth Factor Levels and the Possible Effects of Dexamethasone and Acclimatization Following Re-exposure

**DOI:** 10.3389/fphys.2021.746044

**Published:** 2021-10-21

**Authors:** Craig Winter, Tracy Bjorkman, Stephanie Miller, Paul Nichols, John Cardinal, Peter O’Rourke, Emma Ballard, Fatima Nasrallah, Viktor Vegh

**Affiliations:** ^1^Kenneth Jamieson Department of Neurosurgery, Royal Brisbane and Women’s Hospital, Brisbane, QLD, Australia; ^2^UQ Center for Clinical Research, University of Queensland, Brisbane, QLD, Australia; ^3^School of Mechanical, Medical and Process Engineering, Queensland University of Technology, Brisbane, QLD, Australia; ^4^School of Human Movement Studies, University of Queensland, Brisbane, QLD, Australia; ^5^Queensland Institute of Medical Research, Brisbane, QLD, Australia; ^6^Queensland Brain Institute, University of Queensland, Brisbane, QLD, Australia; ^7^Centre for Advanced Imaging, University of Queensland, Brisbane, QLD, Australia; ^8^ARC Training Centre for Innovation in Biomedical Imaging Technology, Brisbane, QLD, Australia

**Keywords:** VEGF, dexamethasone, acute mountain sickness, blood-brain barrier, acclimatization

## Abstract

**Purpose:** The recognition and treatment of high-altitude illness (HAI) is increasingly important in global emergency medicine. High altitude related hypobaric hypoxia can lead to acute mountain sickness (AMS), which may relate to increased expression of vascular endothelial growth factor (VEGF), and subsequent blood-brain barrier (BBB) compromise. This study aimed to establish the relationship between AMS and changes in plasma VEGF levels during a high-altitude ascent. VEGF level changes with dexamethasone, a commonly used AMS medication, may provide additional insight into AMS.

**Methods:** Twelve healthy volunteers ascended Mt Fuji (3,700 m) and blood samples were obtained at distinct altitudes for VEGF analysis. Oxygen saturation (SPO2) measurements were also documented at the same time-point. Six out of the 12 study participants were prescribed dexamethasone for a second ascent performed 48 h later, and blood was again collected to establish VEGF levels.

**Results:** Four key VEGF observations could be made based on the data collected: (i) the baseline VEGF levels between the two ascents trended upwards; (ii) those deemed to have AMS in the first ascent had increased VEGF levels (23.8–30.3 pg/ml), which decreased otherwise (23.8–30.3 pg/ml); (iii) first ascent AMS participants had higher VEGF level variability for the second ascent, and similar to those not treated with dexamethasone; and (iv) for the second ascent dexamethasone participants had similar VEGF levels to non-AMS first ascent participants, and the variability was lower than for first ascent AMS and non-dexamethasone participants. SPO2 changes were unremarkable, other than reducing by around 5% irrespective of whether measurement was taken for the first or second ascent.

**Conclusion:** First ascent findings suggest a hallmark of AMS could be elevated VEGF levels. The lack of an exercise-induced VEGF level change strengthened the notion that elevated plasma VEGF was brain-derived, and related to AMS.

## Introduction

In the past 5 years, more than 100 million people have traveled to high altitude for work or leisure activities and a significant percentage develop symptoms of high-altitude illness (HAI). The hypobaric hypoxia of high altitude may result in a spectrum of disease, varying from mild symptoms to irreversible cerebral edema and death. Understanding the pathophysiology of HAI can be enhanced by studying the underlying molecular mechanisms, the positive eventual outcome of which is improved management of the condition. Ascending a mountain by an individual can lead to a group of symptoms including headache, nausea, anorexia, lethargy and sleep disturbance, characteristic of acute mountain sickness (AMS).

The etiology of AMS is considered multifactorial and includes mechanisms such as genetic predisposition (MacInnis et al., 2011), dysfunctional cerebral autoregulation, elevated cerebral blood flow, variability in cranio-spinal capacitance, and increased expression of certain hypoxic mediators such as nitric oxide, free radicals, acetyl choline, prostaglandins and vascular endothelial growth factor (VEGF) (Hackett and Roach, 2001; Wilson et al., 2009). VEGF is a 23 kDa protein with a variety of functions, including the induction of endothelial cell proliferation, increased blood vessel permeability, neuroprotection and angiogenesis (Larpthaveesarp et al., 2015). Hypoxia is also a well-recognized stimulus for VEGF release (Juan et al., 2012), possibly via the induction of hypoxia-inducible factor 1 alpha (HIF-1α) (Yeh et al., 2007). It has been suggested that hypobaric hypoxia may induce VEGF release, in turn increased BBB permeability, the drawback of which is unmitigated movement of fluid into the brain. The subsequent development of cerebral vasogenic edema may account for particular AMS symptoms, most likely high-altitude headache.

Based on animal studies, hypoxia results in a significant increase in both VEGF mRNA and protein leading to vascular hyper-permeability (Feng et al., 2010), the application of VEGF antibodies prevents BBB related vascular leakage in hypoxic conditions (Schoch et al., 2002) and exogeneous VEGF results in increased BBB permeability (Jiang et al., 2014). The mechanism of VEGF-induced increased BBB permeability may lie in endothelial barrier disruption following loss of tight junction (TJ) proteins, namely occludin and claudin-5 (Li et al., 2014). To date, human studies have provided conflicting evidence for the relationship between VEGF and high-altitude exposure or AMS. Tissot van Patot et al. (2005) found VEGF levels to increase with altitude and confirmed a positive relation with AMS. Although an increase in VEGF levels has been measured in mountaineers at high altitude by others (Walter et al., 2001; Dorward et al., 2007), neither study found a link with AMS. Some other studies were not even able to establish a VEGF level change at high altitude (Maloney et al., 2000; Palma et al., 2006; Nilles et al., 2009; Schommer et al., 2011), and a well-controlled human study in a simulated hypoxic chamber found, interestingly, a reduction in plasma VEGF levels (Oltmanns et al., 2006). We should point out that high altitude ascent is common across these studies, but the rate of ascent differed. Assimilation of existing findings suggest that VEGF levels do not increase if the rate of ascent is below a certain threshold, i.e., allowing for partial acclimatization. Additionally, people who do not develop AMS tend to have lower VEGF levels, likely a consequence of hypoxia and not AMS.

Dexamethasone is commonly prescribed for both the prophylaxis and treatment of AMS and High-Altitude Cerebral Edema (HACE) (Imray et al., 2010). The exact mechanism of action of dexamethasone is unclear, though may involve changes in calcium-activated potassium channels (Gu et al., 2009), alterations in TJ protein expression such as occludin, claudin-1 and claudin-5 (Sadowski et al., 2010) or the up-regulation of adrenomedullin and HSP-70 (Julian et al., 2011), two anti-inflammatory mediators. *In vitro* studies have confirmed dexamethasone to reduce both hypoxia-induced VEGF and the associated increased BBB permeability (Fischer et al., 2001), and expression of VEGF mRNA and protein from both astrocytes and pericytes of the BBB (Kim et al., 2008). Exercise related increases in VEGF levels have been reported as well (Forsyth et al., 1996; Gagnon et al., 2014; Ross et al., 2014; Vital et al., 2014).

Evidently, high altitude ascent can lead to hypoxia, increased BBB permeability, and in some cases AMS. VEGF levels are known to change with hypoxia. However, it remains unclear whether dexamethasone provides benefits in people who may not have reduced VEGF levels due to a high-altitude ascent. Therefore, we aimed to further explore the intricate relationship between AMS and plasma VEGF during a rapid high-altitude ascent. Our secondary aim was to measure VEGF levels during a second ascent 48 h later to potentially understand the influence of dexamethasone, a medication commonly used for AMS patients.

The second ascent allowed us to potentially comment on the possible effects of dexamethasone on plasma VEGF levels and to observe whether exercise alone causes an elevation of VEGF levels.

## Materials and Methods

The local Ethics Committee provided study approval and methods have previously been described (Winter et al., 2016). Following informed consent, 12 healthy Caucasian volunteers were recruited to ascend Mt Fuji (3,700 m) on two occasions separated by 48 h. The selection of 48 h between ascents was based on best available knowledge at the time and practical limitations. The 48 h gap between the two ascents was chosen to accommodate the availability and study participants. Other limitations included the logistics around traveling to Japan and then to Mt Fuji and back to Australia within a sufficiently short timeframe in view of funding constraints. All participants were healthy (gender ratio of 1:1, age range 22–56) with no recent (less than 12 months) high altitude exposure. Serial blood tests were taken during the ascent and at each bleed point the volunteers documented their oxygen saturation (TuffSat, Datex Ohmeda) and completed the Lake Louise Questionnaire assessment of AMS (Roach et al., 1993). AMS diagnosis was based on a participant experiencing a headache accompanied by at least one other symptom, yielding a score of 4 or more.

The blood tests were taken at the same altitudes for each ascent. On day 1 a baseline sample at 32 m above sea level was taken. The following day (day 2) the group was transported by car (1 h) from the baseline altitude to the start of the trek at an altitude of 1,400 m. The group then ascended to 2,590 m where a blood test was taken, following which we continued upwards to the overnight accommodation at 3,200 m. The next morning (day 3) the group trekked to the summit and remained at this altitude (3,700 m) for 2–3 h, following which another blood sample was taken. A final sample was taken at 2,590 m on descent. No participant took alcohol throughout the study period and regular fluid intake was ensured to prevent dehydration. The second ascent was repeated 48 h later. A second baseline was taken immediately prior to this ascent. Six participants were randomly selected and medicated with Dexamethasone during the second ascent. The standard dose for the prevention or treatment of AMS was prescribed (4 mg twice daily for 21/2 days commencing at the beginning of the second ascent). In summary, blood was extracted from all participants at baseline (day 1), 2,590 m and 3,700 m during ascent on day 2 and 2,590 m during descent on day 3. Two rest days later the ascent and blood tests were replicated.

At each bleed point 12 ml of venous blood (Li-Heparin tubes) was extracted, centrifuged for 3 min at 7,200 rpm (4,400 g) using a portable centrifuge (StatSpin X3) (powered by 2 portable 12V batteries connected to an AC/DC inverter), the plasma removed and placed immediately into dry ice (−80°). The plasma tubes were transported in dry ice to Australia, stored at −80° and assayed for VEGF in duplicate using an R and D Systems Duo ELISA kit as per manufacturers recommendation (intra assay CV < 7%, inter assay CV > 10%). The three high altitude VEGF levels (2,590, 3,700, and 2,590 m on descent) were combined to give a mean average at high altitude (effective altitude of 2,960 m). The mean plasma VEGF values were examined in the presence or absence of AMS. The plasma VEGF level was summarized as mean and the 95% Confidence Interval. A Student’s *t*-test was used to compare VEGF and oxygen saturation between groups. A paired *t*-test was used to compare VEGF and oxygen saturation between altitudes, in addition to analyzing the percentage changes with respect to last measurement, and separately with respect to the first baseline. McNemar’s test was used to compare the presence or absence of AMS between ascents. *F*-test was used to assess differences in group variance. Statistical significance was assumed at *p* < 0.05.

## Results

### First Ascent

**FIGURE 1:**
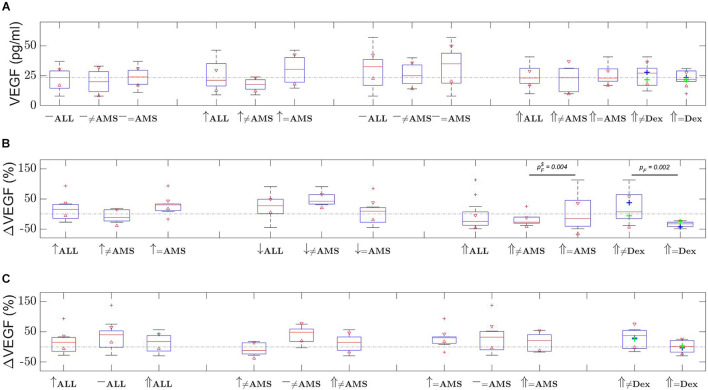
VEGF changes over the time course of the two ascents. Shown are **(A)** distribution of VEGF level measurements, **(B)** relative change in VEGF level with respect to last measurement, and **(C)** relative change in VEGF level with respect to first baseline measurements. Time evolves left to right in **(A,B)**, and in **(C)** dotted line depicts the 0% change level. [Red triangles represent boxplot notches at the 95% confidence level; whiskers correspond with one standard deviation from the mean; red plusses are outliers; green plus is first ascent non-AMS mean, blue plus is AMS mean; ≠ non-AMS or non-dexamethasone (Dex); ↑ first ascent; ↓ descent; ⇑ second ascent; – either first or second baseline; *p*_*F*_ is the *F*-test *p*-value and $ means outlier was removed; the dash-dot line in **(A)** depicts the first baseline mean, in **(B,C)** they show the 0% change, provided as a guide in all cases].

Figure 1A depicts group-by-group VEGF levels, Figure 1B provides the changes in VEGF levels with respect to the last measurement, and Figure 1C is the VEGF level change with respect to first baseline. Note, data provided in Figure 1A is un-paired over the time course. For this reason, relative changes in VEGF levels observed for each individual with respect to the last measurement (Figure 1B) and with respect to first baseline (Figure 1C) have additionally been provided (paired over the time course). The mean plasma VEGF level for all 12 participants during the first ascent was 22.3 pg/ml (95% CI 16.6–28.1) at baseline and 24.9 pg/ml (95% CI 17.4–32.4) at high altitude (*p* = 0.30. During the first ascent 7 participants were deemed to have developed AMS. The mean VEGF level for these 7 participants increased from a baseline 23.9 pg/ml (95% CI 15.8–31.9) to 30.3 pg/ml (95% CI 18.9–41.6) confirming a trend, but not deemed significant (*p* = 0.080). For the remaining 5 participants who did not experience AMS the VEGF decreased from 20.2 pg/ml at baseline (95% CI 7.6–32.8) to 17.4 pg/ml (95% CI 10.3–24.5) at high altitude (*p* = 0.26). It is particularly clear from Figure 1B that VEGF levels for first ascent AMS participants were higher than for the non-AMS group. VEGF levels after the first descent remained high for non-AMS participants, whilst the AMS group had VEGF levels matching all participants at the first baseline.

**FIGURE 2:**
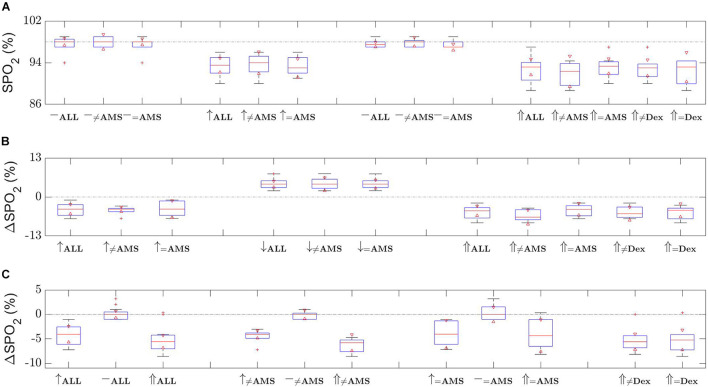
Oxygen saturation (SPO2) changes over the time of the two ascents. Shown are **(A)** distribution of SPO2 level measurements, **(B)** relative change in SPO2 level with respect to last measurement, and (c) relative change in SPO2 level with respect to first baseline measurements. Time evolves left to right in **(A,B)**, and in **(C)** dotted line depicts the 0% change level. [Red triangles represent boxplot notches at the 95% confidence level; whiskers correspond with one standard deviation from the mean; red plusses are outliers; ≠ non-AMS or non-dexamethasone (Dex); ↑ first ascent; ↓ descent; ⇑ second ascent; – either first or second baseline; the dash-dot line in **(A)** depicts the first baseline mean, in **(B,C)** they show the 0% change, provided as a guide in all cases].

Oxygen saturation results show an expected trend, which is around 5% decrease in SPO2 levels at altitude, as shown in Figure 2. For all 12 participants the oxygen saturation reduced from a baseline of 97.8% (95% CI 97.4–98.1) to a mean of 91.6% (95% CI 90.0–93.2) at altitude (*p* < 0.001). The mean oxygen saturation at high altitude for the 7 AMS participants reduced from a baseline of 97.6 (95% CI 97.1–98.1) to 92.0% (95% CI 89.6–94.5), *p* = 0.003, compared to a reduction from 98.0 (95% CI 97.1–98.9) to 91.0% (95% CI 88.0–94.0) for the 5 non-AMS participants, *p* = 0.004. There was no significant difference in the oxygen saturations at baseline or high altitude between the AMS and non-AMS groups with *p*-values of 0.26 and 0.52, respectively.

### Second Ascent

Figure 1 depicts the VEGF levels and percentage changes. Following 48 h of rest the experiment was repeated but with 6 participants (selected randomly) prescribed dexamethasone and 6 non-dexamethasone participants. The mean baseline plasma VEGF level for all 12 participants prior to the second ascent was 30.1 pg/ml (95% CI 20.9–39.3) reducing to 24.2 pg/ml (95% CI 18.5–29.8) at high altitude (*p* = 0.078). For the 6 participants who took dexamethasone the VEGF decreased from 35.2 pg/ml (95% CI 20.6–49.7) to 22.4 pg/ml (95% CI 14.5–30.2) at high altitude (*p* = 0.013) compared to the non-treated 6 participants, where the level increased from 25.0 pg/ml (95% CI 10.0–40.0) to 25.9 pg/ml (95% CI 14.9–36.9) at high altitude (*p* = 0.79). Notably, AMS and non-dexamethasone participants had significantly greater variance in VEGF levels in comparison with non-AMS and dexamethasone prescribed participants. The non-AMS and dexamethasone participants had low VEGF level variability, and mean values were not significantly different (refer to Figure 1B confidence intervals and *F*-test *p*-values).

Figure 2 provides boxplots for the SPO2 changes. During this ascent the reduction in oxygen saturations mirrored the first ascent, as expected, decreasing from baseline to altitude for both the dexamethasone group [baseline 97.3% (95% CI 95.9–98.8), at high altitude 92.2% (95% CI 89.9–94.4), *p* = 0.001] and the non-treated group [baseline 97.8% (95% CI 97.4–98.3), at altitude 92.7% (95% CI 90.4–94.9), *p* = 0.004]. There were no significant differences at either baseline (*p* = 0.41) or at altitude (*p* = 0.70) in oxygen saturations between the dexamethasone and non-treated groups. Additionally, there was no significant difference in the baseline oxygen saturation levels between the two ascents (all participants *p* = 0.62, dexamethasone treated *p* = 0.46, non-treated *p* = 0.36) and no significant difference between the oxygen saturations at high altitude between the two ascents (all participants *p* = 0.075, dexamethasone treated *p* = 0.36, non-treated *p* = 0.14).

When we looked at the incidence of AMS during the second ascent we found that of the seven participants who developed AMS in the first ascent three experienced AMS in the second ascent. Specifically, within the dexamethasone group the three participants who experienced AMS during the first ascent reduced to one person during the second ascent. Similarly, for the non-dexamethasone group the AMS incidence reduced from four participants to two. There was no significant difference in oxygen saturations between AMS and non-AMS participants at baseline (*p* = 0.88) or at altitude (*p* = 0.49). There was no significant difference between oxygen saturation levels at baseline between ascents for the non-AMS (*p* = 0.45) and AMS (*p* = 0.81) participants.

## Discussion

### First Ascent

The seven participants who developed AMS during the first ascent showed a significant increase in plasma VEGF from baseline to high altitude compared to the non-AMS participants. We averaged the three high altitude values (2,590–3,700 and 2,590 m on descent, effectively giving a measurement at 2,960 m) and refer to this as the ‘high altitude’ VEGF level, and this was the value we compared to the baseline level. A high-altitude average was chosen because of the difficulties in assigning significance to one-off measurements which could be influenced by the speed of ascent, the half-life of VEGF and the process of acclimatization.

The 2,590 m value followed a rapid ascent from 1,400 to 2,590 m (1,190 m in 6–8 h) and it is well recognized that HAI (and potentially hypoxia-induced VEGF release) relates to the rate of ascent as well as the final altitude gained. In support of this we found the initial 2,590 m VEGF level was higher than the 3,700 m summit level for some of the participants. In addition, the 3,700 m VEGF level followed a period of overnight acclimatization at 3,200 m after which we ascended to the summit and waited for 2–3 h prior to the sample being taken. The summit VEGF level would have reflected a summation of both acclimatization and a relatively small altitude gain of 500 m (3,200 overnight to the summit at 3,700 m). Similarly, the 2,590 m level during descent was taken within a few hours of maximum hypoxia on the summit and therefore could potentially still reflect a response to the summit altitude.

VEGF has a short half-life (t1/2) (33.7 ± 13.7 min) in normal conditions but the t1/2 of VEGF mRNA increases in hypoxic conditions (up to 3.3 h) and this may also extend to VEGF (Liu et al., 2002). One-off samples may be difficult to interpret in terms of how we relate that level to a specific altitude in the context of a progressive hypoxic stimulus as we ascend. The process of acclimatization also has to be considered. As the body adapts to the decreased availability of oxygen at high altitudes (Ginouves et al., 2008) it causes a respiratory alkalosis, secondary alterations in pH, (bicarbonate) and acid-base balance, all of which are known to influence VEGF release (Shi et al., 2001). The possible role of hypoxic pre-conditioning on VEGF gene up-regulation (Rabie et al., 2011) would likely affect the VEGF level at later time points in the ascent, i.e., the 3,700 and 2,590 m descent levels. For these reasons we decided that an average of the three VEGF high altitude values may better capture a change from baseline, compared to a one-off measurement.

Our baseline VEGF levels are of the same order of magnitude as the literature but the high altitude results differ from some studies, whilst agreeing with others. Tissot van Patot et al. (2005) analyzed VEGF levels from 20 participants who were driven from a relatively low altitude (between 1,370 and 1,645 m) to 4,300 m over a 2-hperiod. Their study design was similar to ours with a rapid ascent, absence of a prolonged period of acclimatization and the conclusion that elevated VEGF levels correlate with AMS. Conversely, Schommer et al. (2011) found that although VEGF did increase with high altitude (4,559 m) they did not find an association with AMS.

In another study, 14 mountaineers ascended to high altitude over an 18–24 h period but no correlation between AMS and VEGF levels were found (Walter et al., 2001). The samples were taken 24 h after the summit altitude was reached, leading to a potential effect of acclimatization. Additionally, the authors diagnosed AMS with a Lake Louise Score of ≥ 5 (in contrast to our study which used a score of 4 or higher for diagnosis). Dorward et al. (2007) flew 42 people to an altitude of 3,650 m followed by 5 days of acclimatization prior to ascending to 5,200 m. The VEGF levels were increased at 5,200 m but no association with AMS was found. Patitucci et al. (2009) established an increase in plasma VEGF at 5,000 m (though no association with AMS) but interestingly this was followed by a decline at an even higher altitude of 8,000 m. The authors suggested that the transient hypoxic induced up-regulation of VEGF expression was followed by down-regulation and this may be part of a complex feedback regulation of the gene HIF 1-α that occurs in prolonged hypoxic conditions (Berra et al., 2006; Ginouves et al., 2008).

Other authors found no association between high altitude and elevated VEGF levels (Maloney et al., 2000; Palma et al., 2006; Nilles et al., 2009) but in each study the samples were delayed, making conclusions problematic. Maloney et al. (2000) investigated VEGF levels at baseline and at 4,200 m in 66 mountaineers. The high-altitude sample was taken following at least 1 day of rest and specimens were kept on ice rather than dry ice or liquid nitrogen. Nine further participants were sampled over 5 days ascending to 4300 m (Palma et al., 2006), and Nilles et al. (2009) also looked at 51 mountaineers during ascent after they had been acclimatising for at least 24 h. Neither study could confirm elevated VEGF levels with altitude or indeed any association with AMS. Most of the studies which did not establish a relationship between high altitude and VEGF level involved a delay at high altitude before VEGF samples were taken and this delay allowed the process of acclimatization to potentially affect the results.

We suggest that the results from our first ascent are robust enough to support an accurate correlation between elevated plasma VEGF and AMS. Importantly, there was no significant difference in the mean oxygen saturations at baseline or high altitude between the AMS and non-AMS groups. The elevated VEGF levels may act on the endothelial cells to open the BBB leading to mild cerebral vasogenic edema and the symptoms of AMS. It is possible that the increased expression of VEGF is the normal response to hypoxia and the variability between individuals is based on a genetic predisposition and that this manifests as the degree of hypoxia that is required to trigger the elevated VEGF expression. Previous investigators have supported the hypothesis that hypoxia-induced VEGF may relate to genetic influences, also citing a possible association between VEGF-A gene and AMS (Ding et al., 2011).

### Second Ascent

At the start of the second ascent half the group (selected at random) were prescribed dexamethasone at a dose commonly used for the prophylaxis and treatment of AMS (Imray et al., 2010). A recent meta-analysis has confirmed the efficacy of dexamethasone for the prevention of AMS (Tang et al., 2014) but there are conflicting results in the literature regarding the specific effects of dexamethasone on VEGF expression. *In vitro* studies have confirmed that dexamethasone will reduce hypoxia-induced VEGF expression (Fischer et al., 2001), decrease VEGF expression in a murine model of hypoxic lung injury (Hegeman et al., 2013) and inhibit hypoxia-inducible factor 1-alpha dependent gene expression (including the VEGF gene) (Wagner et al., 2008). In opposition however, Feng et al. (2011) in an *in vivo* study on rat pups found that dexamethasone provided neuroprotection against hypoxia/ischemic brain injury and that this was associated with an elevation of VEGF expression.

In our study the VEGF level reduced from baseline to high altitude in the 6 dexamethasone participants, in comparison to the non-dexamethasone treated participants where the VEGF levels did not significantly change. The results did not relate to a difference in oxygen saturations at either baseline or high altitude between the two groups. However, drawing any definite conclusions regarding the influence dexamethasone has on plasma VEGF levels becomes problematic when we consider the small sample sizes, and the effect acclimatization may have had on VEGF. The overall incidence of AMS reduced from 7 in the first ascent to 3 in the second ascent but this reduction was not confined to the dexamethasone group. The reduction from 4 participants to 2 in the non-treated group would strongly suggest that the process of acclimatization had occurred.

Adaptations to hypoxia involve the activation of specific genes (Prabhakar and Kline, 2002) and it is possible that 48 h after being upregulated subsequent to the first ascent, downstream gene products resulting in VEGF suppression persisted during the second ascent, making conclusions regarding the isolated effect of the steroid not possible. Additionally, hypoxic preconditioning is the process by which exposure to a moderate hypoxia/ischaemic insult (HI) provides increased resistance to a subsequent episode of HI (Sen et al., 2011; Shao and Lu, 2012) and may also have a genetic underpinning. In experimental terms the first ascent may have provided hypoxic pre-conditioning for our participants prior to the second insult and this may have modified VEGF expression. In essence, the VEGF levels during the second ascent in the dexamethasone group represent the additive effect of dexamethasone, acclimatization and potentially hypoxic preconditioning. That the levels did not change secondary to acclimatization alone (i.e., in the non-treated group) would suggest that dexamethasone did reduce the VEGF levels, but in hindsight it would have been better to have delayed the second ascent for 6 months to rule out any effects of acclimatization. This however was not practically possible given financial restraints and an inability to recruit the same participants 6 months later.

An additional potential influence on peripheral VEGF levels could be the effect of strenuous exertion. The reduced oxygen tension encountered during exercise may cause upregulation of the HIF 1-α gene which stimulates VEGF release (Forsyth et al., 1996) and a resultant elevation of VEGF (Gagnon et al., 2014; Ross et al., 2014). Interestingly, Wahl et al. (2013) describe a significant increase in VEGF at a simulated altitude of 4,000 m only when combined with exercise whilst Vital et al. (2014) found that four papers from a meta-analysis confirmed an exercise-induced increase in VEGF while the remaining six failed to do so. To add to the complexity, a distinction between high and low intensity exercise has been made in the literature such that the former may result in elevated VEGF levels while endurance training does not (Wahl et al., 2011). In our study, VEGF levels increased only in the AMS participants during the first ascent but not in the non-AMS participants, or any of the patients in the second ascent. The results would therefore suggest that elevated peripheral VEGF is not a universal response to exercise, and this would further support the significance of the raised VEGF level in the first ascent correlating with those individuals with altitude sickness. Many of the previous studies do not take the possibility of exercise-induced VEGF into account in their analysis.

Reaching a consensus regarding the role of VEGF in altitude-related illness is difficult given inherent difficulties in comparing results between field studies. The non-standardization of rates of ascent, different final altitudes attained, timing of blood tests, the influence of acclimatization, hydration levels, measuring serum versus plasma samples and accounting for exercise-induced VEGF release often differ between studies. In addition, the diagnosis of AMS using the Lake Louise Questionnaire depends on individuals answering fairly subjective questions, such as rating the severity of headache as mild or moderate. We also note that our results follow an altitude gain of 3,700 m and that a higher altitude could potentially cause a universal VEGF response.

## Conclusion

Changes in VEGF and SPO2 levels were evaluated in 12 participants who ascended Mt Fuji (3,700 m) twice separated by a 48 h rest period. On first ascent participants developing AMS had higher VEGF levels than participants who did not develop AMS. During the second ascent participants who developed AMS on the first ascent tended to have increased VEGF levels compared to non-AMS participants. Participants who took dexamethasone for the second ascent showed VEGF levels similar to those who did not develop AMS during the first ascent, and the AMS and non-dexamethasone groups tended to have similarly elevated VEGF levels. The measured SPO2 levels were unremarkable, other than observing an expected decrease in SPO2 levels at altitude. The lack of exercise-induced VEGF change may suggest that the plasma VEGF was predominantly brain-derived. Insight into the molecular responses by the brain to the hypobaric hypoxia of high altitude may also lead to the improvement in patient management or the development of novel therapeutic strategies for AMS and other hypoxic-ischemic brain injuries, such as subarachnoid hemorrhage and stroke.

## Data Availability Statement

The raw data supporting the conclusions of this article will be made available by the authors, without undue reservation.

## Ethics Statement

The studies involving human participants were reviewed and approved by the HREC Metro North Health Services Ethics. The patients/participants provided their written informed consent to participate in this study.

## Author Contributions

CW: all aspects of research idea and design, interpretation of results, and writing the manuscript. TB: interpretation of results, expert commentary and advice in hypoxic brain injury, and the manuscript review. SM: interpretation of results, advice in hypoxic brain injury, and the manuscript review. PN: interpretation of results and main text corrections. JC: plasma VEGF sample assays and correcting manuscript. FN: expert advice on acquired brain injury and proofreading the manuscript. PO’R and EB: statistical tests and analysis. VV: statistical tests and analysis and proofreading the manuscript. All authors contributed to the article and approved the submitted version.

## Conflict of Interest

The authors declare that the research was conducted in the absence of any commercial or financial relationships that could be construed as a potential conflict of interest.

## Publisher’s Note

All claims expressed in this article are solely those of the authors and do not necessarily represent those of their affiliated organizations, or those of the publisher, the editors and the reviewers. Any product that may be evaluated in this article, or claim that may be made by its manufacturer, is not guaranteed or endorsed by the publisher.

## Abbreviations

HAI: High Altitude Illness
AMS: Acute Mountain Sickness
VEGF: Vascular Endothelial Growth Factor
BBB: Blood-Brain Barrier
HACE: High Altitude Cerebral Edema
HIF-1 α: Hypoxia-inducible factor 1 alpha
HI: Hypoxic-ischaemic insult.
